# CLIMATE CHANGE, AIR POLLUTION, AND MENTAL HEALTH AMONG OLDER KOREAN AMERICAN RESIDENTS IN LOW-INCOME SENIOR HOUSING

**DOI:** 10.1093/geroni/igad104.3227

**Published:** 2023-12-21

**Authors:** Juyoung Park, Yuri Jang

**Affiliations:** University of Southern California, Los Angeles, California, United States; University of Southern California, Los Angeles, California, United States

## Abstract

Given the impact of climate change and air pollution on human health, the present study examined how older individuals’ perceived disruption by extreme weather and air pollution would be linked with mental health. We focused on older Korean Americans living in low-income senior housing and selected anxiety as an indicator of mental health. Data came from surveys with Korean American residents in five low-income senior housing facilities located in the Greater Los Angeles area (n=343, Mean age = 78.4). Anxiety, measured with the GAD-7, averaged 3.38 (SD = 3.77). Perceived levels of disruption caused by extreme weather and air pollution averaged 1.71 (SD = .69) and 1.75 (SD = .72), respectively, out of a range from 1 (not at all) and 4 (very much). Individuals who reported greater disruptions by extreme weather and air pollution had a higher level of anxiety. Given the high correlation between the two types of disruption (r = .56, p < .001), we tested separate regression models of anxiety. Each variable of the perceived disruption by extreme weather (β = .18, p < .001) and air pollution (β = .18, p < .001) was found to have a significant impact on anxiety. Although limited by the use of a small sample of convenience restricted to one ethnic group in one geographic location, our findings suggest the potential mental health impact of climate change and air pollution and call attention to intervention strategies.

